# The anticancer effect of γ-irradiation synthesized selenium nanoparticles stabilized in β-glucan on HepG2 cell proliferation *via* apoptosis induction and cell cycle arrest

**DOI:** 10.1039/d5na00733j

**Published:** 2025-12-10

**Authors:** Duc Trong Tran, Thanh Vu Nguyen, Thi Dung Nguyen, Van Linh Nguyen, Quang Luan Le

**Affiliations:** a Biotechnology Center of Ho Chi Minh City Ho Chi Minh City Vietnam lequangluan@gmail.com; b Ho Chi Minh University of Natural Resources and Environment Ho Chi Minh City Vietnam

## Abstract

The product of selenium nanoparticles (SeNPs) stabilized in water-soluble yeast β-glucan (SeNPs/β-glucan) was successfully synthesized by γ-ray irradiation on a scale of 3 liters per batch. The analysis results of its transmission electron microscopy (TEM) image showed that SeNPs in the product were spherical with an average actual particle size of about 63.3 nm, while dynamic light scattering (DLS) analyses indicated that the average hydrodynamic particle size of the product was about 93.5 nm with a narrow distribution and negative zeta potential value (−10.1 mV). In addition, the results also showed the hydrodynamic particle size and size distribution of the product slightly increased after storage for 60 days at 4 °C, whereas a more pronounced increase was observed when stored at room temperature (25 °C). Besides, the structural characteristics of SeNPs/β-glucan were also comprehensively analyzed using X-ray diffraction (XRD), Raman spectroscopy and Fourier transform infrared (FTIR) spectroscopy to confirm the crystal structure of the Se nanoparticles and their interaction with β-glucan molecules. The anticancer effects of SeNPs/β-glucan on the liver cancer cell line (HepG2) were also investigated and the obtained results demonstrated that SeNPs/β-glucan strongly inhibited the proliferation of HepG2 cells with a half maximal inhibitory concentration (IC_50_) of about 6.5 ppm, while its IC_50_ on the normal cell line (L929) was found to be 48.3 ppm, indicating very low cytotoxicity. The selectivity index (SI) value of the product was determined to be around 7.4, indicating selective toxicity toward cancer cells. Furthermore, apoptosis assays demonstrated that SeNPs/β-glucan induced apoptosis and inhibited the proliferation of HepG2 cells by triggering cell cycle arrest in the S and G2/M phases in a dose-dependent manner. These findings provide a theoretical foundation and experimental evidence supporting the potential applications of SeNPs/β-glucan in the food and pharmaceutical fields.

## Abstract 2

The γ-ray irradiation synthesized SeNPs/β-glucan with high stability, safe for normal cells and selective cytotoxicity against liver cancer cells. The product is a potential candidate to produce ingredient with cancer prevention and therapy functions.

## Graphical abstract

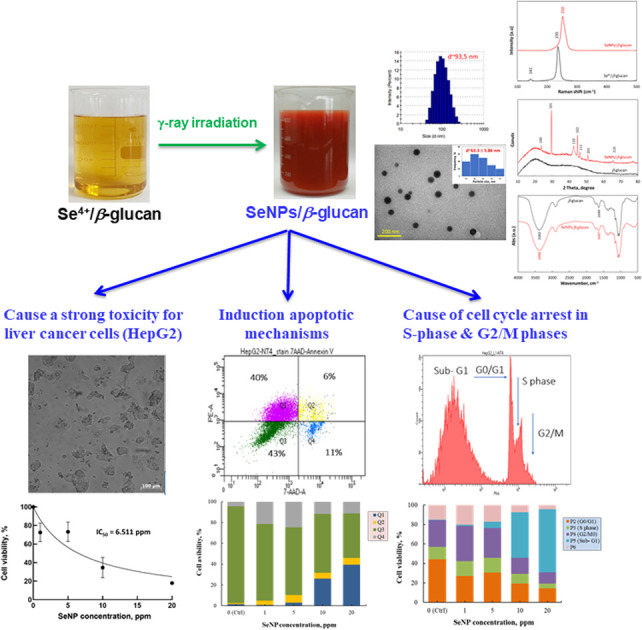

## 1. Introduction

Nanotechnology is a cutting-edge field that focuses on the design, synthesis, and analysis of particle structures ranging from 1 to 100 nm in size. This advanced technology significantly impacts various industries and sectors, including nanomedicine, nanoelectronics, biomaterials, energy production, and consumer products. Nanoparticles possess unique physical, chemical, and biological properties that differ significantly from their bulk counterparts, largely due to their small size and large surface area. Selenium (Se) is an essential trace element, which is crucial for human and animal health, impacting numerous biological functions such as antioxidant, anticancer, antiviral, and antibacterial activities, and enhancing immune response. The significant biological activities of selenium are mainly due to its incorporation into selenoproteins, which play vital roles in cellular processes like redox regulation and thyroid hormone metabolism. However, the bioavailability and toxicity of Se are heavily influenced by its chemical form.

Selenium nanoparticles (SeNPs) have emerged as a prominent option due to their superior bioavailability, enhanced bioactivity, and reduced toxicity relative to other forms of selenium, both inorganic and organic.^1,2^ SeNPs exhibit remarkable properties, making them suitable for various biological applications.^3^ They interact with biological molecules such as selenoproteins, selenocysteine, and selenomethionine, playing a crucial role in biological systems. Several studies have demonstrated the efficacy of SeNPs in combating severe diseases like cancer and diabetes.^4,5^ The biological functionality and toxicity of SeNPs are influenced by factors such as size, morphology, and surface chemical composition. Previous research has indicated that smaller SeNPs with a spherical shape and smooth surface are more effective in biological applications due to their higher cellular uptake and interaction with biomolecules.^3–6^

Various methods have been employed to synthesize SeNPs from ionic Se solutions, including biological,^7^ chemical,^8^ and irradiation methods.^9,10^ Each method presents distinct advantages and challenges. Biological methods, involving microorganisms or plant extracts, offer an eco-friendly and cost-effective approach. Chemical methods allow precise control over the synthesis process but may involve toxic reagents. Irradiation methods, particularly γ-ray irradiation from a Co-60 source, stand out due to several advantages: they can be performed at room temperature, ensure high purity with simplified purification, allow control of particle size by adjusting the dose or dose rate, and are suitable for large-scale production.^9,11^

The synthesis of SeNPs often involves the use of stabilizers to control particle size and prevent aggregation. Traditional stabilizers include polymers and surfactants, but water-soluble and low molecular weight yeast β-glucans have not been commonly used. Exploring the potential of low molecular weight yeast β-glucans as stabilizers in SeNP synthesis could be a promising avenue for future research and applications. β-Glucans are known for their immunomodulatory properties and biocompatibility, which could enhance the therapeutic potential of SeNPs.

Numerous studies have highlighted the potential of SeNPs in cancer prevention, both as anticancer agents and as carriers for delivering anticancer drugs. When combined with anticancer drugs, SeNPs can effectively treat cancer due to their high bioavailability and low toxicity to normal cells, while being highly toxic to cancer cells. These nanoparticles, typically ranging in size from 10 to 100 nm, can penetrate cancerous tissues and selectively destroy them.^12^ Importantly, SeNPs do not penetrate healthy cells, even when their particle size is very small (about 2–6 nm).^13^ The mechanism by which SeNPs inhibit cancer cells primarily involves inducing apoptosis and/or disrupting the cell cycle at various stages. SeNPs can induce apoptosis through intrinsic and extrinsic pathways, increasing the production of reactive oxygen species (ROS) and activating caspase enzymes, leading to programmed cell death.^14–16^

Liver cancer is one of the leading causes of cancer-related death worldwide. In Vietnam, liver cancer has a high incidence and mortality rate, accounting for the majority of cancer-related deaths.^17^ Current cancer treatment regimens, including surgery, radiotherapy, and chemotherapy, non-specifically target healthy cells, leading to adverse reactions and side effects.^18^ Conventional chemotherapy faces limitations such as poor solubility of anticancer drugs, lack of selectivity, and multidrug resistance.^19^ These challenges necessitate the development of new treatment strategies that actively target cancer cells with minimal impact on healthy tissues.

Therefore, this study aimed to synthesize the SeNPs/β-glucan product by γ-ray irradiation with a scale of 3 liters per batch and evaluated its inhibitory effects on HepG2 cell proliferation. Furthermore, the inhibitory mechanisms in cancer cells, including apoptosis induction and cell-cycle disruption, were investigated to provide valuable insights into the potential application of SeNPs in liver cancer treatment, contributing to the development of more effective and targeted cancer therapies. By exploring the synthesis, characterization and biological activity of SeNPs/β-glucan synthesized by γ-ray irradiation, the present study seeks to advance the understanding and application of the product in liver cancer therapy.

## 2. Experimental section

### 2.1. Materials and reagents

Yeast water-soluble β-glucan (1–3, 1–6) with a molecular weight of approximately 25 kDa was a gift from Biomaterials and Nano Technology Department, Biotechnology Center of Ho Chi Minh City, Vietnam. Sodium selenite was purchased from Sigma (St. Louis, MO). MTT (3-(4,5-dimethylthiazol-2-yl)-2,5-diphenyl tetrazolium bromide), fetal bovine serum (FBS), dimethyl sulfoxide, Dulbecco's modified Eagle medium (DMEM), trypsin, penicillin–streptomycin, K_2_S_2_O_8_, KI, and NaOH were obtained from Sigma. The apoptosis kit was obtained from BD Biosciences. The HepG2 (human liver carcinoma) and L929 (normal cell) cell lines were obtained from the Food Biotechnology Department, Biotechnology Center of Ho Chi Minh City, Vietnam.

### 2.2. Preparation of SeNPs by γ-ray irradiation

For synthesis of SeNPs/β-glucan, the solutions containing 80 ppm Se^4+^ and 2% water-soluble yeast β-glucan with a total volume of 3 liters were irradiated at doses from 4 to 10 kGy with a dose rate of 10 kGy h^−1^ using a GC5000 Gamma Co-60 Chamber (BRIT, India).

### 2.3. Characterization of SeNPs synthesized by γ-ray irradiation

The Se^4+^ ion contents in Se^4+^/β-glucan solutions before and after irradiation at various doses were analyzed by centrifuging at 111 400×*g* for 30 min using an Optima MAX-XP ultracentrifuge (Beckman Coulter, USA) for separation of SeNPs. The remaining Se^4+^ ion content in supernatants collected after centrifugation was analyzed by a spectrophotometric method using Azure B for chromogenic regents.^20^

The hydrodynamic particle size and zeta potential of SeNPs in irradiated solutions were analyzed with a Malvern Zetasizer model ZEN5600 (Malvern, UK) using Zetasizer Software V.7.12, while the actual particle size was characterized by transmission electron microscopy (TEM) using a JEM-1010 (JEOL, Japan).

The crystal lattice structure of SeNPs was characterized using an X-ray diffractometer (XRD) D8 Advance Eco (Bruker, Germany) with parafocusing Bragg–Brentano geometry using Cu Kα radiation (*λθ* = 1.5418 Å, *U* = 40 kV, and *I* = 30 mA) and a LabRAM HR Evolution confocal Raman microscope system (Horiba, Japan) using a 785 nm laser wavelength and LabSpec 6.5.1 software was used to analyze data. Moreover, the functional groups present in SeNPs/β-glucan were analyzed using Fourier-transform infrared spectroscopy (FTIR) (FTIR-4700, Shimadzu, Japan).

The stability of the SeNPs/β-glucan product stored at 4 °C and room temperature (25 °C) for 60 days also was evaluated *via* the increase of the average hydrodynamic particle size using the Malvern Zetasizer.

### 2.4. *In vitro* cytotoxicity assay

The cytotoxic effects of SeNPs/β-glucan on HepG2 cells and fibroblast L929 cells were evaluated using the MTT method.^21^ HepG2 cells were seeded into flat-bottomed 96-well DMEM culture plates at a density of 1 × 10^4^ cells per well. The cells were treated with SeNPs/β-glucan at concentrations of 1, 5, 10, and 20 ppm, while the 0 ppm sample (only water without SeNPs/β-glucan) served as a control sample. All treatments were incubated for 24 hours and performed in triplicate. After incubation, 50 µL of MTT solution at a concentration of 0.4 mg mL^−1^ was added to each well and incubated for 4 hours. The resulting crystals were dissolved in dimethyl sulfoxide. The amount of formazan salt was determined by measuring the absorbance at 570 nm using a microplate reader. Optical densities were used to calculate the percentage viability in treated cells compared to untreated control cells. The half maximal inhibitory concentration (IC_50_) values were determined using polynomial regression analysis of dose–response curves (log[inhibitor] *vs.* normalized response) fitted with a four-parameter logistic model using GraphPad Prism 8.4.3 software. The IC_50_ of the product on normal cells was also determined on fibroblast L929 cells. The IC_50_ values of SeNPs/β-glucan in both cell lines were used to determine the selectivity index (SI) using the equation SI = IC_50-L929_/IC_50-HepG2_. Compounds are classified as highly selective if the SI value exceeds 3 and less selective if the SI value is <3.^22^

### 2.5. Apoptosis assay

To determine cell mortality rates and apoptosis induced by SeNPs/β-glucan, cells were stained with Annexin V/7-amino-actinomycin and analyzed by flow cytometry. HepG2 cells were seeded onto 6-well plates and allowed to adhere. After the cells reached 70% confluence (1 × 10^6^ cells per tube), they were treated with SeNPs at concentrations of 0 (the control) 1, 5, 10, and 20 ppm at 37 °C and 5% CO_2_ for 24 hours. Subsequently, the cells were collected and washed with PBS before being resuspended in 200 µL of 1× annexin-binding buffer. The cells were then incubated at room temperature with staining solution (5 µL Annexin V-FITC and 5 µL 7-amino-actinomycin) in the absence of light. Following a 20 min incubation, samples were immediately analyzed *via* flow cytometry to determine the apoptosis rates of SeNPs/β-glucan-treated HepG2 cells. Annexin V staining was detected as green fluorescence and 7-amino-actinomycin as red fluorescence.

### 2.6. Cell-cycle analysis

Cell cycle perturbations were assessed using flow cytometry to measure the proportion of cells in different phases. The effect of SeNPs/β-glucan on cell cycle phase distribution was evaluated using propidium iodide (PI) staining. HepG2 cells were harvested 24 hours after treatment with SeNPs/β-glucan at concentrations of 0 (the control supplied with distilled water), 1, 5, 10, and 20 ppm. The cells were then collected and fixed in ice-cold 70% ethanol overnight and stored at −20 °C until PI staining. Ethanol-suspended cells were centrifuged at 1500 rpm for 4 min and washed in PBS with 0.1% bovine serum albumin (BSA) to remove residual ethanol. The cell pellets were resuspended in 1 mL of a fluorochrome solution (0.1% Triton X-100, PBS, DNAse-free RNAse, and PI) and incubated at 20 °C for 30 min. Finally, cell cycle profiles were obtained using a BD FACScan flow cytometer (BD Biosciences Accuri C6 Plus, USA) and data were analyzed using Cell Quest Pro software.

### 2.7. Statistical analysis

Statistical comparisons were conducted using the ANOVA test. The least significant difference (LSD) at a 5% probability level was used to compare the mean values. The results were expressed as means ± standard error (SE) and *P*-values less than 0.05 were considered significant.

## 3. Results and discussion

### 3.1. Characteristics of SeNPs/β-glucan synthesized by γ-ray irradiation

**Fig. 1:**
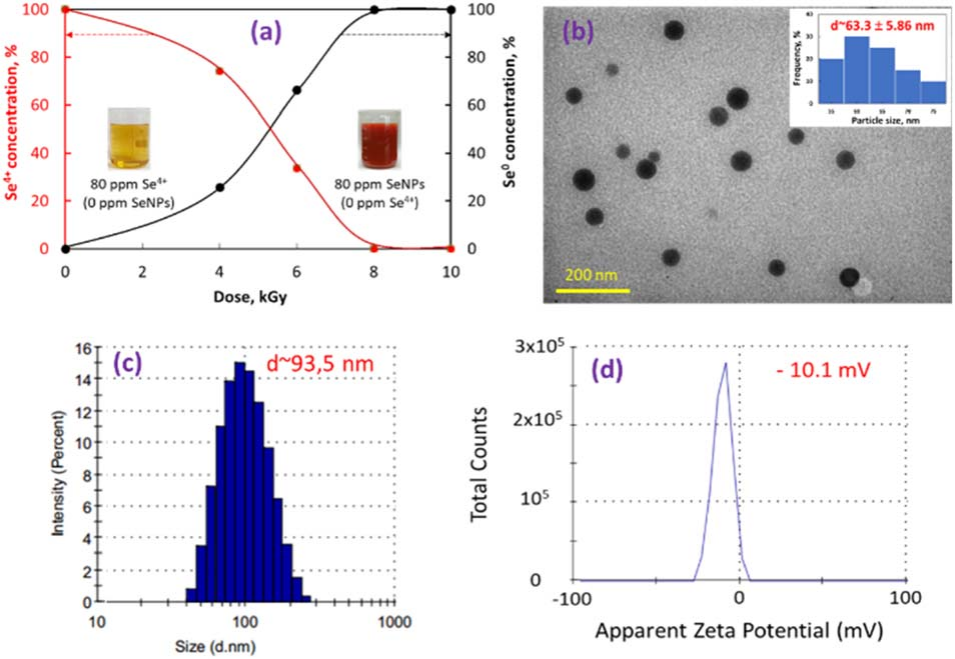
The effect of dose on γ-ray irradiation synthesis (a), TEM image and actual particle size (b), hydrodynamic particle size (c) and zeta potential value (d) of SeNPs stabilized in water soluble β-glucan.

The results from Fig. 1a indicated that the contents of Se^4+^ ions in samples irradiated at 8 and 10 kGy were completely reduced to Se^0^ and the dose required for complete reduction of 80 ppm Se^4+^ ions to SeNPs was found to be 8 kGy. In addition, Fig. 1a also shows the change in the color of Se^4+^/β-glucan solution from light yellow to orange-red SeNPs/β-glucan solution after irradiating at 8 kGy. These results are in good agreement with those reported by Duy *et al.*^9^ and Hien *et al.*^10^ on the usage of gamma Co-60 ray irradiation for synthesis of SeNPs stabilized in oligochitosan and dextran, respectively. The as-synthesized SeNPs were thoroughly characterized by TEM (Fig. 1b) and the results from the TEM image confirmed the spherical shape of SeNPs with an actual particle size of about 63.3 nm. On the other hand, the analytical results from dynamic light scattering in Fig. 1c and d showed that the average hydrodynamic particle size and zeta potential of SeNPs/β-glucan solution were about 93.5 nm and −10.1 mV, respectively. These characteristics of the current product are almost similar to those of the SeNPs/β-glucan sample prepared on a scale of 100 mL in our previous paper and in good agreement with those reported by Souza *et al.* as well.^6,23^ In addition, the results from Fig. 1b and c also showed a Gaussian distribution with a narrow distribution for both actual and hydrodynamic particle sizes, which indicated a uniformity in the size of nanoparticles.

**Fig. 2:**
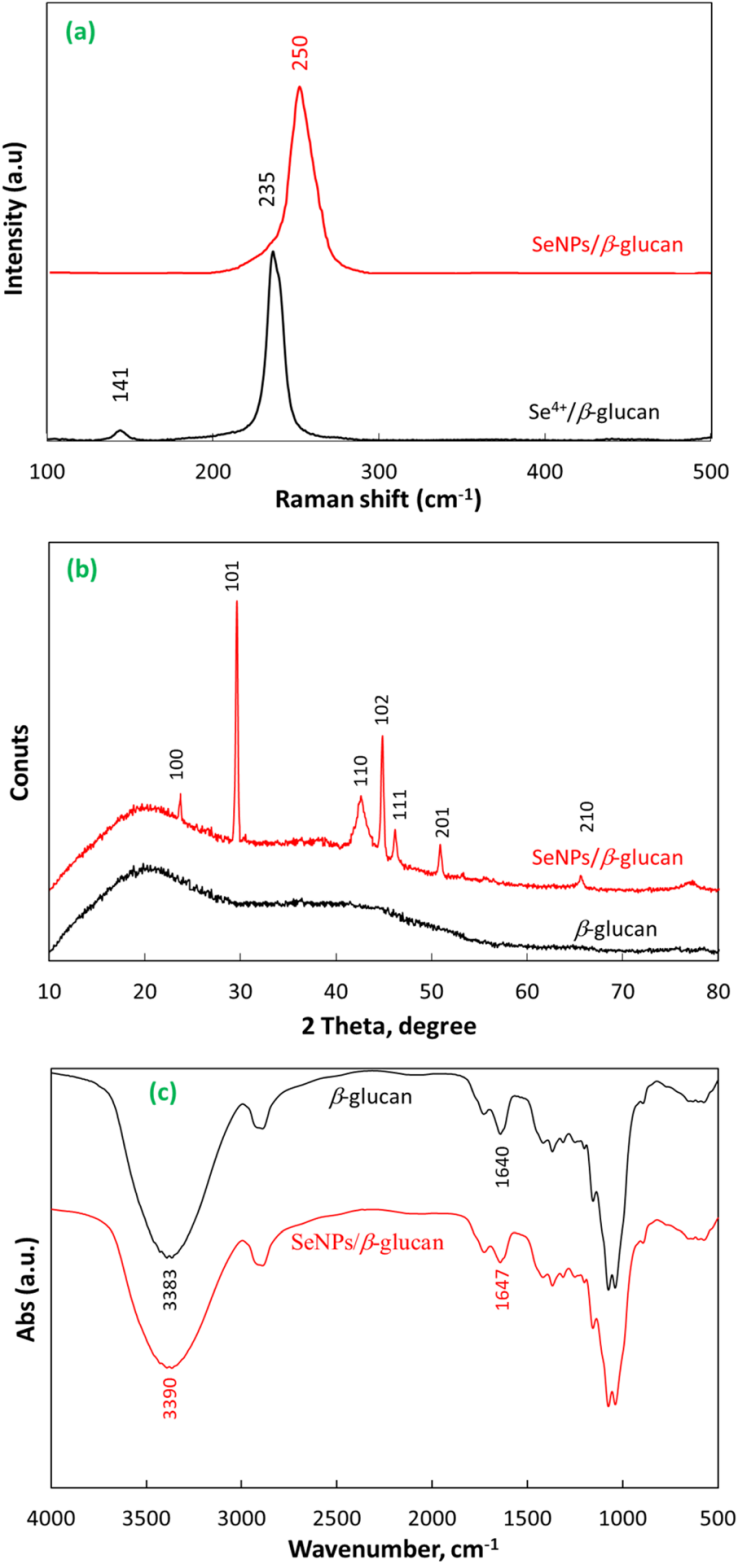
The Raman spectra (a), XRD patterns (b), and FTIR spectra (c) of SeNPs/β-glucan synthesized by γ-ray irradiation.

Raman spectroscopy has been used to examine structural changes in materials including the conversion of Se^4+^ ions to selenium atoms (Se^0^) due to its sensitivity in chemical identification.^24^ According to Minaev *et al.*, non-crystalline substances can be broadly classified into two types: vitreous and ultra-dispersive substances.^25^ In the present study, the Raman spectrum of the Se^4+^/β-glucan solution before irradiation in Fig. 2 exhibited two characteristic peaks at 141 and 235 cm^−1^ attributed to photon vibrational modes of the Se^4+^ ions. These results are in good agreement with those reported by Minaev *et al.* that photon vibrations assigned for vitreous selenium appeared at 140 and 235 cm^−1^.^25^ After radiation at 8 kGy, the photon vibrations were no longer present in the spectrum of SeNPs/β-glucan, while a new photon vibration peak appeared at 250 cm^−1^ attributed to the α-monoclinic selenium crystalline form.^25,26^ These results revealed that γ-ray irradiation caused a structural transformation of the selenium species from vitreous selenium (Se^4+^) to α-monoclinic selenium (Se^0^).

XRD analysis was used to determine the crystallinity of SeNPs after irradiation. The results in Fig. 2b indicated that SeNPs exhibited diffraction peaks at 23.7° (100), 29.5° (101), 42.7° (110), 44.8° (102), 46.2° (111), 51° (201), and 65.3° (210) attributed to the crystal structure of SeNPs and these results are in good agreement with those of SeNPs/dextran reported by Hien *et al.*^9^

The recorded FTIR spectra in Fig. 2c showed that the main peaks of β-glucan at around 3383, 2986, 1640, 1156, and 890 cm^−1^ corresponding to the bonds of –OH, –CH, –C=O, C–O–C, and β-d-glucan were all present in the SeNPs/β-glucan spectrum. Moreover, the peaks at 3383 and 1640 cm^−1^ assigned to the OH group vibrations of β-glucan (Fig. 2c) shifted to higher wavenumbers of 3390 and 1647 cm^−1^ in the case of SeNPs/β-glucan. Our results are in good agreement with those of SeNPs/oligochitosan reported by Duy *et al.*^10^

The uniformity in the size and shape of SeNPs, as well as their crystalline structure, are critical factors influencing their biological activities and applications. The small size and spherical morphology observed in TEM analysis suggest enhanced cellular uptake and interaction, which are advantageous for biomedical applications such as drug delivery and imaging. In conclusion, the synthesized SeNPs demonstrate suitable physicochemical properties for biomedical applications.

**Fig. 3:**
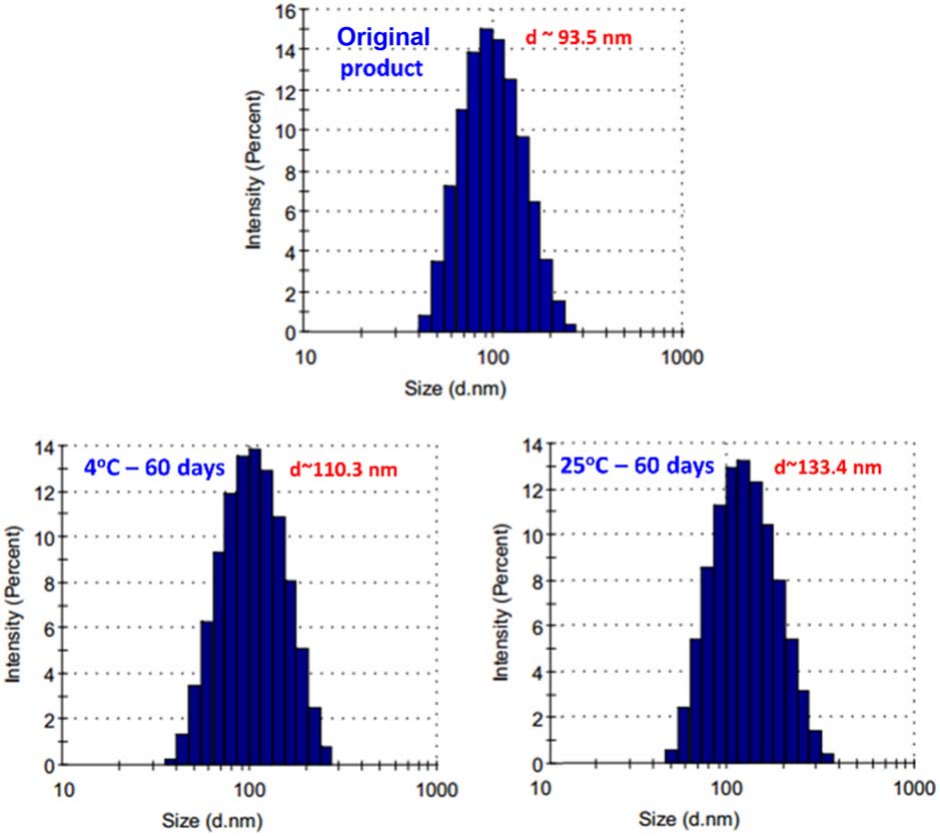
The hydrodynamic particle size of the SeNPs/β-glucan product after storage for 60 days at 4 °C and room temperature (25 °C).

The results from Fig. 3 indicated that the size of hydrodynamic particles in the SeNPs/β-glucan product only slightly increased from 93.5 to about 110.3 nm after 60 days of storage at 4 °C, while the increase of particle size from 93.5 to approximately 133.4 nm was observed when stored at room temperature (25 °C). These results are consistent with the study by Duy *et al.*, which reported that SeNP sizes in SeNP/oligochitosan increased more rapidly at 27 °C than at 4 °C.^10^ According to Bai *et al.*, such particle size changes can be attributed to the Brownian movement of SeNPs causing agglomeration.^27^

### 3.2. Cytotoxicity assay of SeNPs/β-glucan on HepG2

**Fig. 4:**
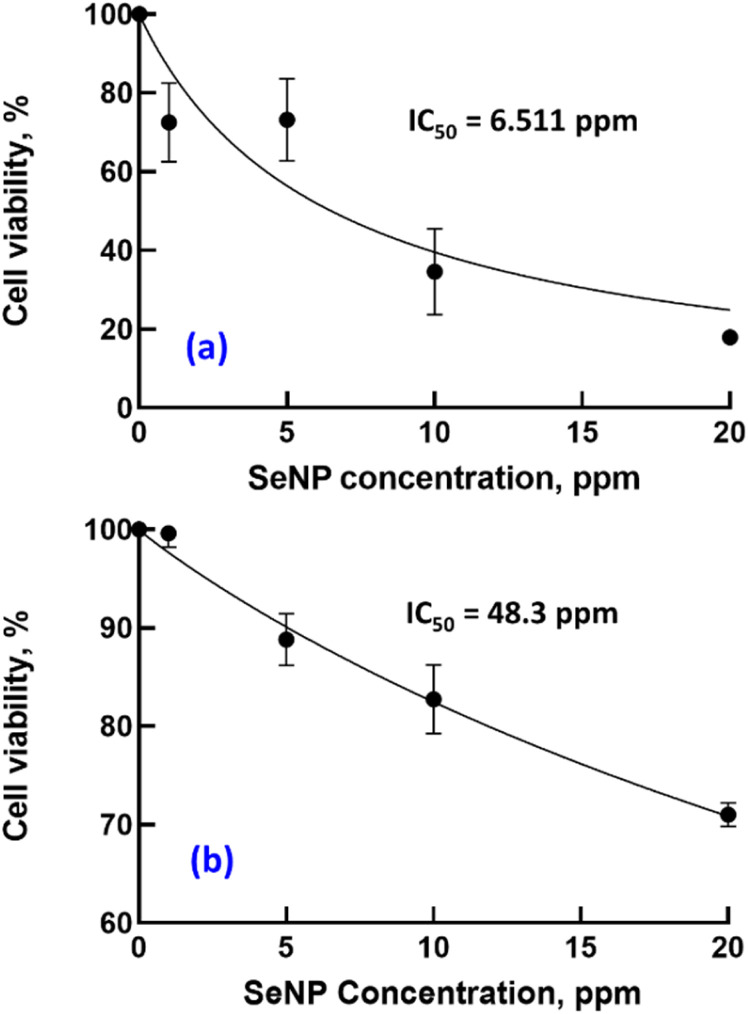
The IC_50_ values of SeNPs/β-glucan for HepG2 (a) and L929 (b) cell lines.

**Fig. 5:**
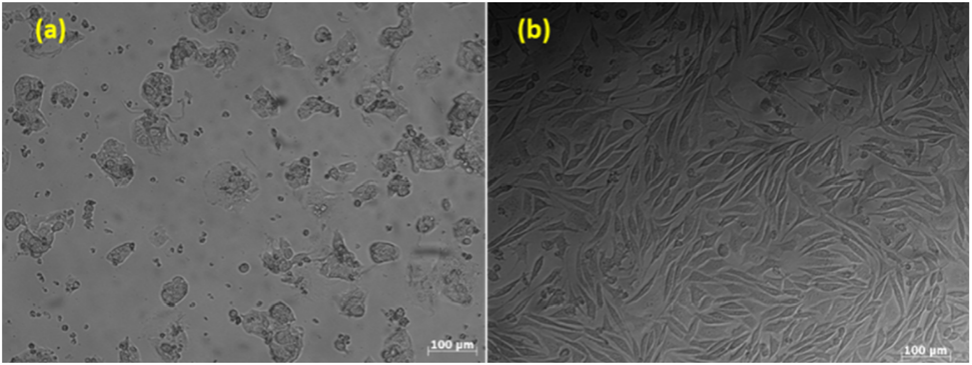
Morphology of cancer cells (a) and normal cells (b) after treatment with SeNPs/β-glucan at a concentration of 20 ppm.

The evaluation of the effects of SeNPs/β-glucan on liver cancer cell proliferation revealed a concentration-dependent response, suggesting a mode where the proliferation decreased with increasing concentrations. The IC_50_ value was determined to be 6.5 ppm (Fig. 4), indicating strong cytotoxic activity of SeNPs against HepG2 cells (Fig. 5a). This initial finding highlights the potential of SeNPs/β-glucan in both preventing and treating cancer. Notably, this IC_50_ value is lower than those reported in recent studies. Particularly, Alam *et al.*^28^ reported an IC_50_ of 25 ppm for SeNPs on HepG2 cells, while Indumathy *et al.*^29^ reported that only 33.7% cell viability of the HepG2 cell line was observed with 30 ppm SeNPs. In addition, Salah *et al.* demonstrated an IC_50_ value of 8.87 µg mL^−1^ against HepG2 cells for SeNPs synthesized by gamma irradiation and selective cytotoxicity toward cancer cells over normal cells.^30^ These comparisons highlight the enhanced potency of SeNPs/β-glucan synthesized by γ-ray irradiation in this study against the HepG2 cell line.

According to Cannella *et al.*, L929 cells are suitable for use as normal cells to undergo a cytotoxicity test by MTT assay, as they are sensible and able to respond well to any cytotoxic substance in culture media.^31^ The results from Fig. 4 and 5a demonstrated a higher tolerance of the L929 fibroblast cell line to SeNPs/β-glucan with survival rates over 70% at concentrations ranging from 1 to 20 ppm and an IC_50_ value of 48.3 ppm. This suggests a selective cytotoxic effect of SeNPs/β-glucan on cancer cells while exhibiting lower toxicity toward normal cells, as indicated by an SI value of 7.4. The SI is a metric used to gauge the relative safety of a compound by comparing its toxicity to cancer cells *versus* normal cells. Krug *et al.*^32^ demonstrated that SeNPs accumulated in the liver of mice in the *in vitro* experiment, indicating the targeted therapeutic potential of SeNPs/β-glucan for liver cancer. Furthermore, a higher SI value indicates a greater selectivity in targeting cancer cells while minimizing adverse effects on normal cells, aligning with the criteria proposed by Weerapreeyakul *et al.*^33^ and emphasized by Indrayanto *et al.*^34^ for evaluating compounds with potential in cancer treatment. The interaction of SeNPs with healthy cells may differ from their interaction with cancer cells, which could be attributed to factors such as size or surface properties. Healthy cells and their membranes have specific size and surface charge characteristics that can affect the uptake of nanoparticles. Therefore, nanoparticles need to be sufficiently small to pass through cell membranes or be taken up *via* endocytosis, and their surface characteristics, such as charge or coating, can significantly influence this process. Additionally, surface coatings or functional groups on SeNPs can further affect their cellular uptake, with coatings designed to enhance biocompatibility or targeting potentially influencing how readily specific types of cells take up nanoparticles.

### 3.3. Apoptosis assay of SeNPs/β-glucan on HepG2

Apoptosis, a programmed cell death process in multicellular organisms, is characterized by distinct morphological and molecular changes such as nuclear fragmentation, chromatin condensation, cell shrinkage, and DNA fragmentation. In this study, the Annexin V-FITC/7-AAD double staining method was employed to assess apoptosis in HepG2 cells treated with varying concentrations (1–20 ppm) of SeNPs/β-glucan for 24 hours.

**Fig. 6:**
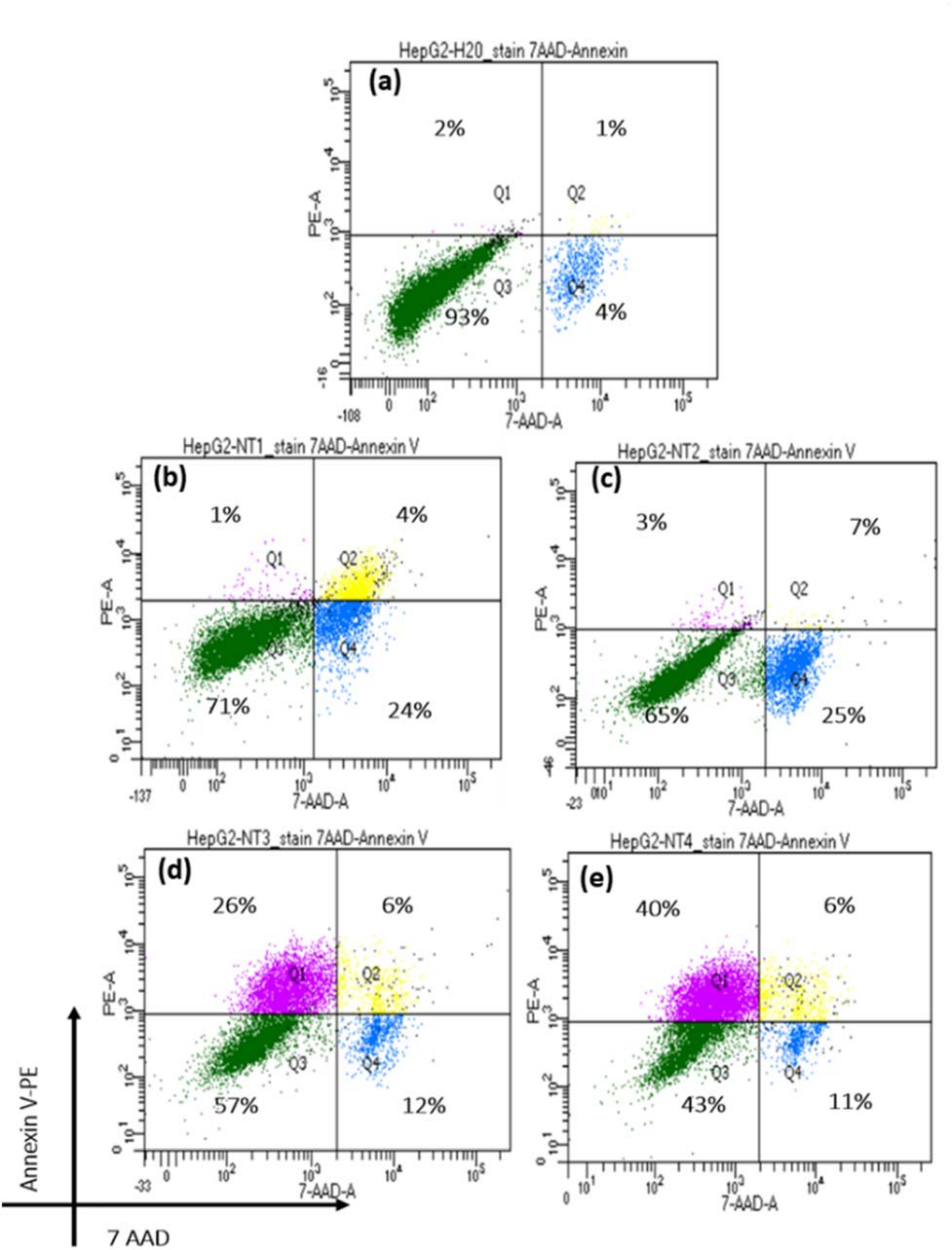
Flow-cytometry analyses of SeNPs/β-glucan with dual staining of Annexin V-PE and 7AAD. Each graph consists of 4 quadrants: 1st quadrant Q1 (early apoptosis cells), 2nd quadrant Q2 (late apoptosis cells), 3rd quadrant Q3 (living cells), and 4th quadrant Q4 (necrosis cells). The graphs include the following treatments: (a–e) SeNPs/β-glucan treatments at concentrations of 0 (control), 1, 5, 10 and 20 ppm, respectively.

**Fig. 7:**
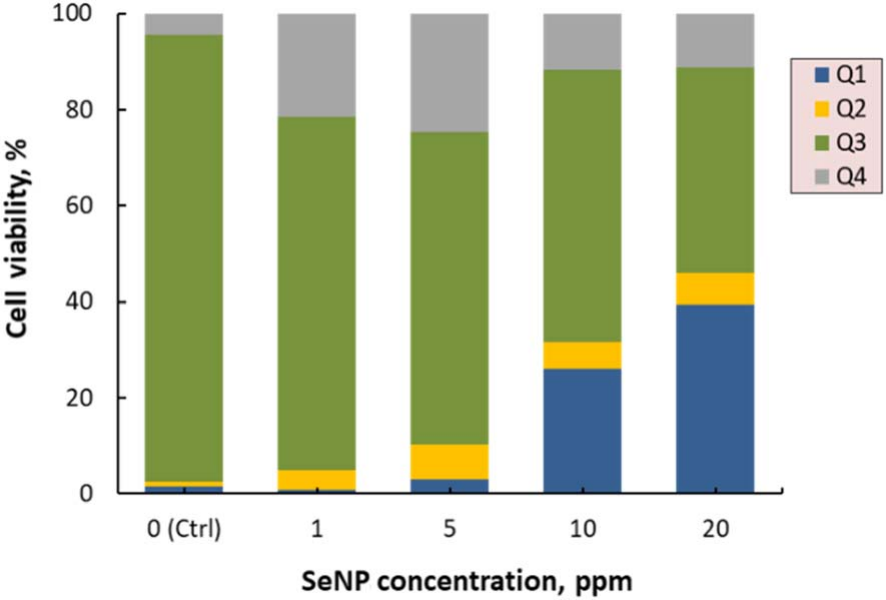
Apoptosis induced by SeNPs/β-glucan with dual staining of Annexin V-PE and 7AAD. Q1 (early apoptosis cells), Q2 (late apoptosis cells), Q3 (living cells), and Q4 (necrosis cells).

Fig. 6 and 7 show that the control (water-treated) samples predominantly consist of Annexin V-PE and 7AAD non-binding cells (live cells), comprising approximately 92% of the population. Conversely, the proportions in Q1, Q2, and Q4 are minimal (<5%). Treatments with increasing concentrations of SeNPs/β-glucan from 1 to 20 ppm resulted in a gradual decrease in the live cell population (Q3) with increasing treatment doses. Notably, at the highest concentration (20 ppm), almost no live cells were detected. However, at lower concentrations (1 and 5 ppm), a significant proportion of cells was observed in Q4 (7AAD positive and Annexin V-PE negative), indicating late apoptosis or necrosis. In contrast, the Q1 region (7AAD negative and Annexin V-PE positive, indicating early apoptosis) was nearly absent (<5%) at 1 ppm, but increased from 26 to 66% starting from 5 ppm treatment, becoming predominant at 20 ppm.

These findings indicate that the SeNPs/β-glucan product synthesized by γ-ray irradiation can induce apoptosis in liver cancer cells in a concentration-dependent manner. As the concentration of SeNPs/β-glucan increases, more cells enter the early apoptosis stage (Q1), aligning with similar observations by Cui *et al.*^15^ Similar apoptotic induction has been reported for SeNPs stabilized in β-glucan by He *et al.* and Tang *et al.*, although our irradiation-synthesized product exhibits stronger early-apoptotic dominance at comparable concentrations.^16,35^ Notably, He *et al.* included β-glucan alone as a control and found negligible cytotoxic or cell cycle effects on HepG2 cells compared with the pronounced activity of SeNPs and SeNPs stabilized in β-glucan.^16^ This supports that SeNPs are the principal bioactive component, while β-glucan primarily functions as a stabilizer. In the present study, the irradiation-synthesized SeNPs/β-glucan product demonstrated a stronger early-apoptotic dominance at comparable concentrations, further highlighting the effectiveness of this system. Moreover, the apoptosis-inducing activity of SeNPs has been suggested to be mediated through both death receptor and mitochondrial pathways. This suggests that SeNPs/β-glucan may inhibit liver cancer cells by promoting programmed cell death, underscoring their potential application in cancer therapy.

### 3.4. Cell-cycle analysis of SeNPs/β-glucan on HepG2

**Fig. 8:**
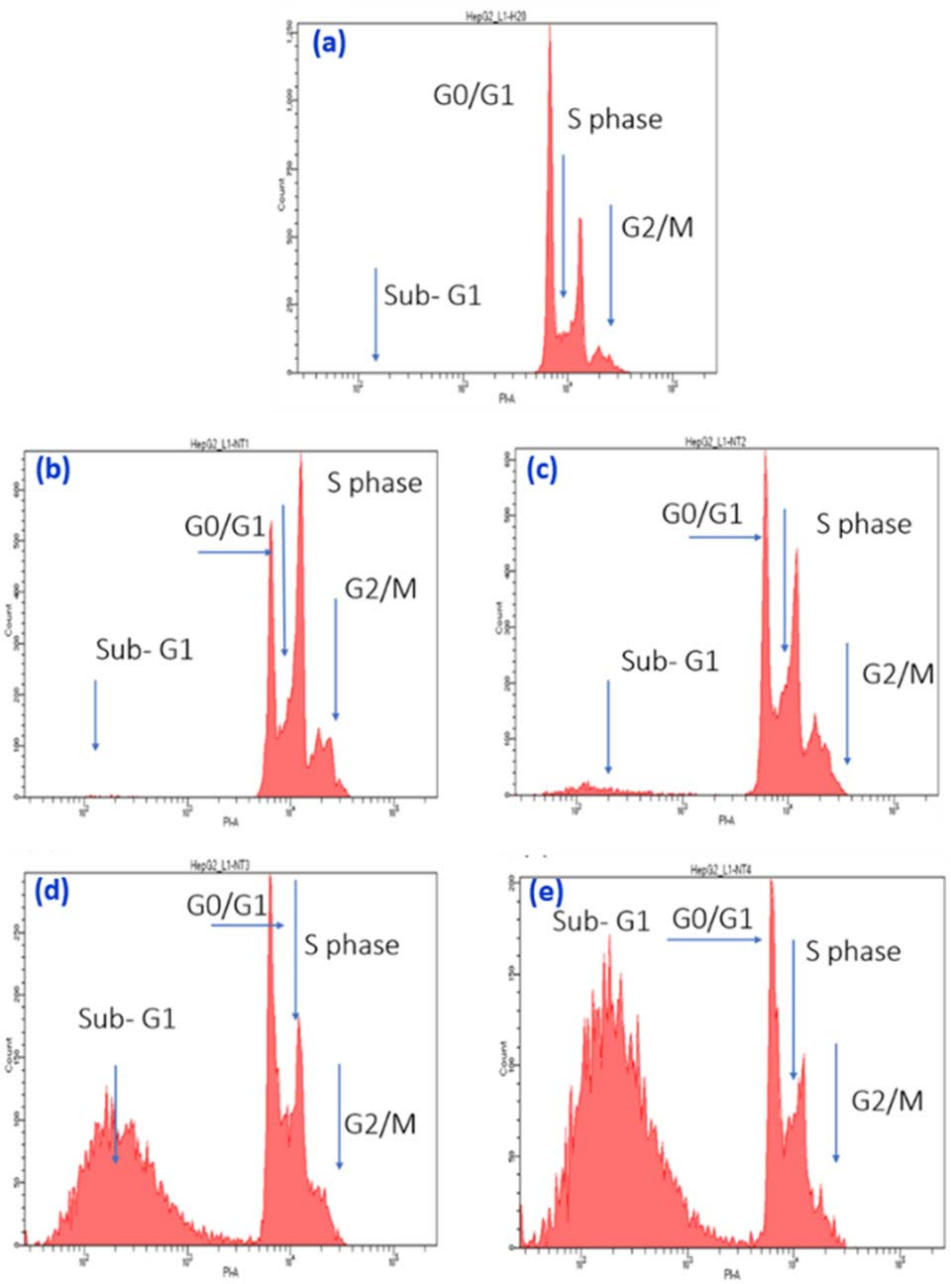
Flow cytometry analysis of cell cycle phase distribution in the liver cancer cell line HepG2. (a–e) SeNPs/β-glucan treatments at concentrations of 0 (control), 1, 5,10, and 20 ppm, respectively.

**Fig. 9:**
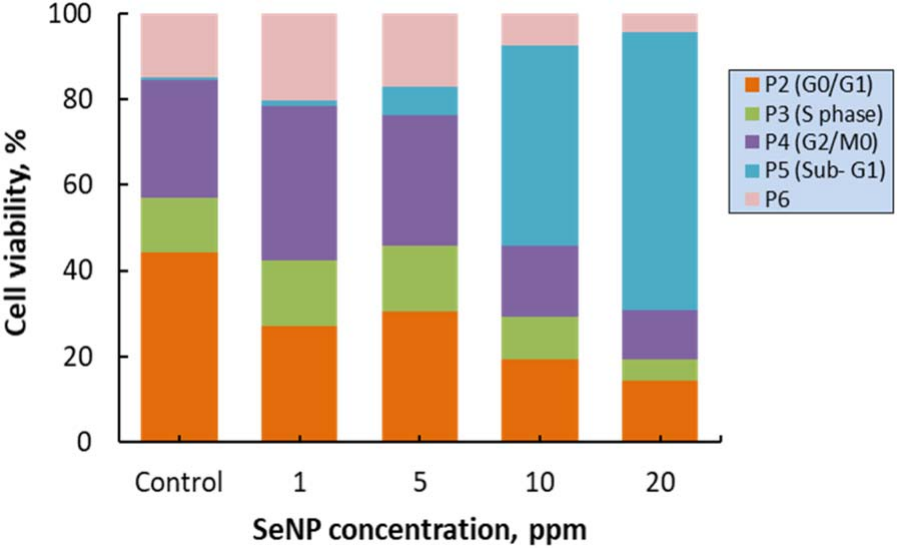
Cell cycle arresting potential of SeNPs/β-glucan in HepG2 cells.

One of the fundamental requirements for cell growth and proliferation is the efficient progression of the cell cycle, which includes four distinct phases: G1 (gap 1), S (DNA synthesis), G2 (gap 2), and M (mitosis). Dysregulation of the cell cycle is a hallmark of cancer cells, stemming from disrupting regulatory mechanisms that govern cell division. Consequently, targeting cell cycle progression is a crucial strategy in current anti-cancer drug development. In this study, flow cytometry was employed to investigate the impact of SeNPs/β-glucan on the cell cycle progression of HepG2 cells treated with varying concentrations (1–20 ppm) for 24 hours, assessed *via* PI staining (Fig. 8 and 9). As depicted in the figures, the percentage of cells in the sub-G1 phase significantly increased following 24 hours of SeNPs/β-glucan treatment. Specifically, exposure to SeNPs/β-glucan at 10 and 20 ppm resulted in a notable increase of the sub-G1 cell population by 65 and 99% (*p* < 0.05), respectively, compared to the control sample (1%). The sub-G1 cell population represents cells with fragmented DNA, a characteristic marker of apoptosis. Moreover, this increase in the sub-G1 cell population was accompanied by a corresponding reduction in cells in the S-phase and G2/M phases of the cell cycle. These findings suggest that SeNPs/β-glucan inhibits HepG2 cell proliferation by inducing cell cycle arrest in the S-phase and G2/M phases, thereby promoting accumulation of cells in the sub-G1 phase, which is indicative of DNA fragmentation and late-stage apoptotic or necrotic cells with low PI intensity. The effect of SeNPs/β-glucan on HepG2 cells was potent and dose-dependent.

Numerous studies have investigated the anticancer effects of SeNPs; however, there remains a gap in research specifically addressing their impact on liver cancer cells, particularly those synthesized *via* irradiation and stabilized with β-glucan. Lopez-Heras *et al.*^36^ investigated the apoptotic and cell cycle effects of chitosan-stabilized SeNPs (Ch-SeNPs) on HepG2 liver cancer cells. Their study demonstrated that Ch-SeNPs induced apoptosis and disrupted the cell cycle of HepG2 cells, leading to decreased G0/G1 phase cells and increased S-G2/M phase cells, indicating S phase cell cycle arrest induced by Ch-SeNPs. These nanoparticles induced cell cycle perturbations and disrupted normal cell division processes, underscoring their potential in cancer therapy. In conclusion, the SeNPs/β-glucan product synthesized by γ-ray irradiation induced apoptotic mechanisms and caused cell cycle arrest in the S-phase and G2/M phases, resulting in an increased proportion of cells in the sub-G1 phase of the cell cycle in human liver cancer HepG2 cells. These findings highlight the potential therapeutic application of SeNPs/β-glucan in liver cancer treatment, warranting further investigation into their mechanistic pathways and clinical efficacy.

## 4. Conclusion

An SeNPs/β-glucan product with an actual hydrodynamic particle size of about 93.5 nm with a spherical shape and narrow distribution was successfully synthesized by γ-ray irradiation on a scale of 3 liters per batch. The product demonstrated significant cytotoxic effects on HepG2 cells, while exhibiting minimal toxicity towards the normal L929 cell line. Notably, this study identified the mechanism of HepG2 cell inhibition through apoptosis induction and disruption of cell cycle progression in the S and G2/M phases in a dose-dependent manner. This mechanistic insight highlights a novel aspect of SeNPs/β-glucan, distinguishing this product from previously reported SeNPs. The demonstrated selective cytotoxicity against cancer cells, combined with the biocompatible stabilizing role of β-glucan and the advantages of γ-ray irradiation synthesis, underscores the potential of SeNPs/β-glucan as a safe and effective candidate for liver cancer therapy.

## Author contributions

Duc Trong Tran: writing – original draft, methodology, investigation, software, formal analysis, data curation and visualization; Thanh Vu Nguyen: writing – original draft, methodology, software and formal analysis; Quang Luan Le: writing – review & editing, methodology, visualization, validation, conceptualization, supervision, project administration and funding acquisition; Thi Dung Nguyen: writing & editing, methodology, investigation, formal analysis, data curation, conceptualization and funding; Van Linh Nguyen: writing original draft, investigation, software and formal analysis. Duc Trong Tran and Thanh Vu Nguyen are co-first authors and contributed equally.

## Conflicts of interest

There are no conflicts to declare.

## Data Availability

Data for this article, including the structure and bioactivity of SeNPs/β-glucan are available at SeNPs-beta glucan. See DOI: https://doi.org/10.57760/sciencedb.33184.
